# Influence of association state and DNA binding on the O_2_-reactivity of [4Fe-4S] fumarate and nitrate reduction (FNR) regulator

**DOI:** 10.1042/BJ20140169

**Published:** 2014-09-08

**Authors:** Jason C. Crack, Melanie R. Stapleton, Jeffrey Green, Andrew J. Thomson, Nick E. Le Brun

**Affiliations:** *Centre for Molecular and Structural Biochemistry, School of Chemistry, University of East Anglia, Norwich Research Park, Norwich NR4 7TJ, U.K.; †Department of Molecular Biology and Biotechnology, University of Sheffield, Sheffield S10 2TN, U.K.

**Keywords:** cluster conversion, dimerization, DNA regulation, fumarate and nitrate reduction (FNR) regulator, iron–sulfur cluster, O_2_-sensor, CRP, cAMP receptor protein, FNR, fumarate and nitrate reduction (regulator), *Nm*-FNR, *Neisseria meningitidis* FNR

## Abstract

The fumarate and nitrate reduction (FNR) regulator is the master switch for the transition between anaerobic and aerobic respiration in *Escherichia coli*. Reaction of dimeric [4Fe-4S] FNR with O_2_ results in conversion of the cluster into a [2Fe-2S] form, via a [3Fe-4S] intermediate, leading to the loss of DNA binding through dissociation of the dimer into monomers. In the present paper, we report studies of two previously identified variants of FNR, D154A and I151A, in which the form of the cluster is decoupled from the association state. *In vivo* studies of permanently dimeric D154A FNR show that DNA binding does not affect the rate of cluster incorporation into the apoprotein or the rate of O_2_-mediated cluster loss. *In vitro* studies show that O_2_-mediated cluster conversion for D154A and the permanent monomer I151A FNR is the same as in wild-type FNR, but with altered kinetics. Decoupling leads to an increase in the rate of the [3Fe-4S]^1+^ into [2Fe-2S]^2+^ conversion step, consistent with the suggestion that this step drives association state changes in the wild-type protein. We have also shown that DNA-bound FNR reacts more rapidly with O_2_ than FNR free in solution, implying that transcriptionally active FNR is the preferred target for reaction with O_2_.

## Abstract 2

DNA-binding of [4Fe-4S] FNR and amino acid substitutions that decouple the dependence of the association state on the cluster are shown to have no effect on the overall cluster conversion mechanism but have a significant effect on reaction kinetics.

## INTRODUCTION

*Escherichia coli* is a metabolically versatile chemoheterotroph, capable of growth on various substrates under various oxygen tensions. Under anaerobic conditions, fumarate or nitrate, among others, can replace O_2_ as the terminal electron acceptor [1]. Optimal switching from one respiratory pathway to another is thus a key requirement for this flexibility. In *E. coli*, the fumarate and nitrate reduction (FNR) transcriptional regulator is responsible for sensing environmental levels of O_2_ and controlling the switch to anaerobic respiration [2–5].

FNR is a member of the cAMP receptor protein (CRP)-FNR superfamily of homodimeric transcriptional regulators, which consist of an N-terminal sensory domain and a C-terminal DNA-binding domain. Although a high-resolution structure is not yet available, the homology of FNR with the structurally characterized CRP [6], together with extensive biochemical data, has enabled a structural model to be proposed in which an O_2_-sensing [4Fe-4S] cluster is located in the N-terminal domain, co-ordinated by four cysteine residues (Cys^20^, Cys^23^, Cys^29^ and Cys^122^) [7–9]. In the absence of O_2_, monomeric (~30 kDa) FNR acquires a [4Fe-4S]^2+^ cluster, triggering a conformational change at the dimerization interface that leads to the formation of homodimers (~60 kDa) and site-specific DNA binding [10,11]. Upon exposure to O_2_, the [4Fe-4S]^2+^ cluster is converted into a [2Fe-2S]^2+^ form [8,12] via a mechanism involving a [3Fe-4S]^1+^ intermediate [13,14]. Cluster conversion results in a re-arrangement of the dimer interface, leading to monomerization [10]. In this respect, *E. coli* and closely related FNR proteins are unique. Other members of the CRP-FNR family typically remain dimeric, irrespective of the presence of their analyte [15], showing that the conformational changes that switch the DNA affinity are not associated with a monomer–dimer equilibrium.

The FNR variant D154A exhibits an increased tendency to dimerize and, as a result, is constitutively active (a so-called FNR* variant) [3,16,17]. This substitution falls within a region (residues 140 to 159) analogous to the dimerization helix of CRP. Moore and Kiley [17] showed that the negatively charged side chain of Asp^154^ is oriented towards the dimer interface, where inter-subunit charge repulsion is proposed to inhibit dimerization before cluster acquisition. Thus, insertion of the [4Fe-4S]^2+^ cluster apparently causes shielding of the negative charge of Asp^154^, thereby facilitating dimerization. Removal of the negatively charged side chain by substitution also alleviates the repulsion, even in the absence of a cluster, leading to a predominantly dimeric form [17].

Ile^151^, a residue also in the dimerization helix, plays a critical role in the shielding of the negative charge of the Asp^154^ side chain upon cluster acquisition. The I151A variant, in which isoleucine is replaced by a residue with a significantly shortened hydrophobic side chain, appears to be less able to shield the negative charge of Asp^154^ after cluster acquisition. As a result, I151A exists as a monomer, even in the presence of a [4Fe-4S]^2+^ cluster [17].

The requirement of a dimeric form of FNR for high-affinity DNA binding, together with the O_2_-sensitive [4Fe-4S] to [2Fe-2S] cluster transformation that controls the FNR monomer–dimer equilibrium, implies that the reactivity of the cluster will be linked to the association state in such a way that anything that influences the monomer–dimer equilibrium will also affect the cluster O_2_-reactivity. The D154A and I151A FNR variants provide a means to test this possibility.

Site-specific binding of dimeric [4Fe-4S] FNR to DNA represents another interaction that could affect potentially the cluster conversion reaction. DNA binding could, through induced conformational changes, influence the accessibility of O_2_ to the cluster, or perhaps the precise arrangement of co-ordinating cysteine residues that may influence the redox properties of the cluster. An early investigation of the effect of DNA binding on the *E. coli* FNR reaction with O_2_ concluded that it had essentially no effect [18]. However, a 34-bp dsDNA oligomer containing only the consensus FNR-binding site was used, and thus some features contributing to the nucleoprotein complex that are more remote from the core binding site might not have been revealed. Furthermore, the study used an iron chelator to monitor cluster conversion, and it is known that external iron chelators can have a significant effect on the kinetics of cluster conversion [13]. This re-examination of the effect of DNA binding was further prompted by studies of *Neisseria meningitidis* FNR (Nm-FNR), which showed that DNA binding has a significant effect on the cluster conversion reaction, increasing the initial O_2_ reaction rate, although the conversion of the intermediate [3Fe-4S]^1+^ cluster into the [2Fe-2S]^2+^ cluster was slowed significantly, thereby prolonging the period over which FNR remains transcriptionally active [19].

In the present paper, we report *in vivo* studies of D154A and I151A FNR variants that confirm the importance of FNR dimerization for transcriptional activity and showed that the rate of transcriptional response, dependent on [4Fe-4S] cluster incorporation or [4Fe-4S] into [2Fe-2S] conversion, is not significantly affected by, respectively, pre-loading of D154A FNR on to DNA or its inability to monomerize following O_2_ exposure. We have also reported *in vitro* studies that show that D154A FNR closely resembles the wild-type protein in the spectroscopic properties of its cluster and also exhibits similar reactivity towards O_2_. I151A FNR, in contrast, exhibits differences in its cluster properties and reactivity with O_2_, resulting principally in an enhanced rate of [3Fe-4S]^1+^ into [2Fe-2S]^2+^ conversion. We have also reported studies of the reactivity of wild-type FNR in the presence of DNA, which revealed a 2-fold enhancement of the rate constant for the initial reaction with O_2_ and an apparent enhancement of [3Fe-4S]^1+^ into [2Fe-2S]^2+^ conversion, relative to the DNA-free control. The implications of this work, with respect to the coupling of cluster reactivity with association state and the O_2_-sensing function of FNR *in vivo*, are discussed.

## MATERIALS AND METHODS

### Plasmid construction

The FNR protein was overproduced initially as a GST–FNR fusion from the expression plasmid pGS572 [20]. Equivalent expression plasmids encoding C16A/C122A GST–FNR (pGS2257a) and I151A GST–FNR (pGS2252) were constructed by site-directed mutagenesis of pGS572 using the QuikChange® system (Stratagene). A previously constructed GST–FNR D154A expression plasmid (pGS771) [21] was used as the template for synthesis of the GST–FNR C122A/D154A expression plasmid (pGS2267), again using the QuikChange® protocols. Plasmid pGS422 contains a 343-bp DNA fragment containing consensus FNR-binding site (FF-41.5, TTGATGTACATCAA) located between the EcoRI and HindIII restriction sites of pUC13 [22]. For *in vivo* transcription studies, pBR322 derivatives encoding the following FNR variants under the control of the *fnr* promoter as HindIII/BamHI fragments were used: pGS196 (FNR), pGS385 (FNR C122A), pGS2405 (FNR D154A), pGS2401 (FNR C122A/D154A) ([23] and the present study). The authenticity of all plasmids was confirmed by DNA sequencing.

### *In vivo* expression studies

*E. coli* JRG6348 is an *fnrlac* deletion strain with a chromosomal copy of *lacZ* fused to a semi-synthetic FNR-dependent promoter (FF-41.5). JRG6348 was transformed by plasmids encoding the indicated FNR variants. Aerobic cultures were grown with shaking (250 rev./min) at 37°C in conical flasks containing LB broth and ampicillin (200 μg·ml^−1^) up to 10% of their total volume until exponential phase was reached (a *D*_600_ of 0.3–0.4). Anaerobic cultures were grown in sealed bottles containing LB broth supplemented with ampicillin at 37°C until exponential phase was reached (*D*_600_ of 0.15–0.25). In some experiments, aerobic cultures were grown and then transferred into sealed bottles, and vice versa, to study the dynamics of FNR switching *in vivo*. Samples were removed as indicated and β-galactosidase activities were measured according to the Miller protocol [24]. In all cases, pre-cultures were grown under the same conditions (i.e. either aerobic or anaerobic) as the initial conditions used in the experiments. Each experiment was performed at least three times.

### Purification of *in vivo* and *in vitro* assembled [4Fe-4S] FNR

Aerobic cultures of *E. coli* BL21λDE3 containing pGS572 (GST–FNR), pGS771 (D154A GST–FNR), pGS2252 (I151A GST–FNR) or pGS2257a (C16A/C122A GST–FNR) were grown, and GST–FNR overproduction was initiated by the addition of IPTG (0.4–1 mM). *In vivo* cluster assembly of GST–FNR fusion proteins was promoted under anaerobic conditions, as described previously [25]. GST–FNR fusion proteins were isolated anaerobically using buffer A (25 mM Hepes, 2.5 mM CaCl_2_, 100 mM NaCl and 100 mM NaNO_3_, pH 7.5), and FNR was cleaved from the fusion protein using thrombin, as described previously [25]. *In vitro* cluster assembly was carried out in the presence of NifS, as described previously [25].

### Isolation of [2Fe-2S] FNR

Samples of [2Fe-2S] FNR were prepared freshly by combining an aliquot of [4Fe-4S] FNR (100 μl of typically ≥700 μM cluster) with an aliquot (500 μl) of buffer B (10 mM potassium phosphate, 400 mM KCl and 10% glycerol, pH 6.8) containing dissolved atmospheric O_2_. The sample was gently mixed in the presence of air for 2 min before being returned to the anaerobic cabinet (Belle Technology) and immediately passed down a desalting column equilibrated with buffer A (PD10, GE Healthcare) to remove low-molecular-mass species (e.g. O_2_ and Fe^3+/2+^) before use.

### Spectroscopy

Absorbance measurements were made with a Jasco V550 UV–visible spectrophotometer under anaerobic conditions via coupling to an anaerobic cabinet via a fibre optic interface (Hellma). CD measurements were made with a Jasco J-810 spectropolarimeter. EPR measurements were made with an X-band Bruker EMX EPR spectrometer equipped with an ESR-900 helium flow cryostat (Oxford Instruments). Spin intensities of paramagnetic samples were estimated by double integration of EPR spectra using 1 mM Cu(II) and 10 mM EDTA as the standard.

### Kinetic measurements

Kinetic measurements of the reaction of [4Fe-4S] FNR proteins were performed at 25°C by combining different aliquots (2 ml of total volume) of aerobic and anaerobic buffer A or buffer B, as described previously [13]. Reaction was initiated by the injection of an aliquot of native or reconstituted [4Fe-4S] FNR (5 μM), and the mixture was stirred throughout. To investigate the effect of DNA on the reaction kinetics, FNR samples were incubated with a 1.2-fold molar excess of FNR-binding sites in the form of pGS422 (pU13 FF-41.5) or a 345-bp PCR product containing the FNR-binding site amplified from pGS422 (FF-41.5) in buffer C (20 mM Tris/HCl and 5% glycerol, pH 8.0), incubated at 25°C for 30 s, before the addition of buffer C containing dissolved atmospheric O_2_ (219.2 μM). The dead time of mixing was ~5 s. Changes in absorbance (*A*_420_) were used to monitor cluster conversion. Milligram quantities of pGS422 were purified using Gigakit (Qiagen) according to the manufacturer's instructions and resuspended in anaerobic buffer C. For kinetic measurements of the reaction of [2Fe-2S] forms of the FNR variants, protein solutions were mixed with excess buffer B containing dissolved atmospheric O_2_ (~30 μM [2Fe-2S], ~120 μM O_2_, final concentration), incubated at 19°C and monitored at *A*_420_.

### Data analysis

FNR cluster conversions were followed under pseudo-first-order conditions (with oxygen in excess) by measuring absorbance changes at 420 nm. Datasets were fitted either to a single exponential or, where single exponential fits were not satisfactory, to a double exponential function, as described previously [13,14]. Observed rate constants (*k*_obs_) obtained from the fits (in the case of fitting to a double exponential function, the rate constant of the first reaction phase was used) were plotted against the corresponding initial concentration of O_2_ to obtain the apparent second-order rate constant. Fitting of kinetic data was performed using Origin (version 8, Origin Labs) and Dynafit [26]. Estimates of errors for rate constants are represented as±S.E.M. values.

### Quantitative methods

FNR protein concentrations were determined using the method of Bradford (Bio-Rad Laboratories), with BSA as the standard, and a previously determined correction factor of 0.83 [27] for apo-FNR. Iron and acid-labile sulfide contents were determined, as described previously [28,29]. Based on the analyses, both native and reconstituted [4Fe-4S] FNR samples exhibited ε_405 _values of ~16220 M^−1^·cm^−1^, in close agreement with previously reported values [13,18]. The concentration of dissolved atmospheric O_2_ present in buffer solutions was determined by chemical analysis according to the method of Winkler [30].

## RESULTS

### Iron–sulfur cluster incorporation and dimerization are required for FNR transcriptional activity *in vivo*

**Figure 1:**
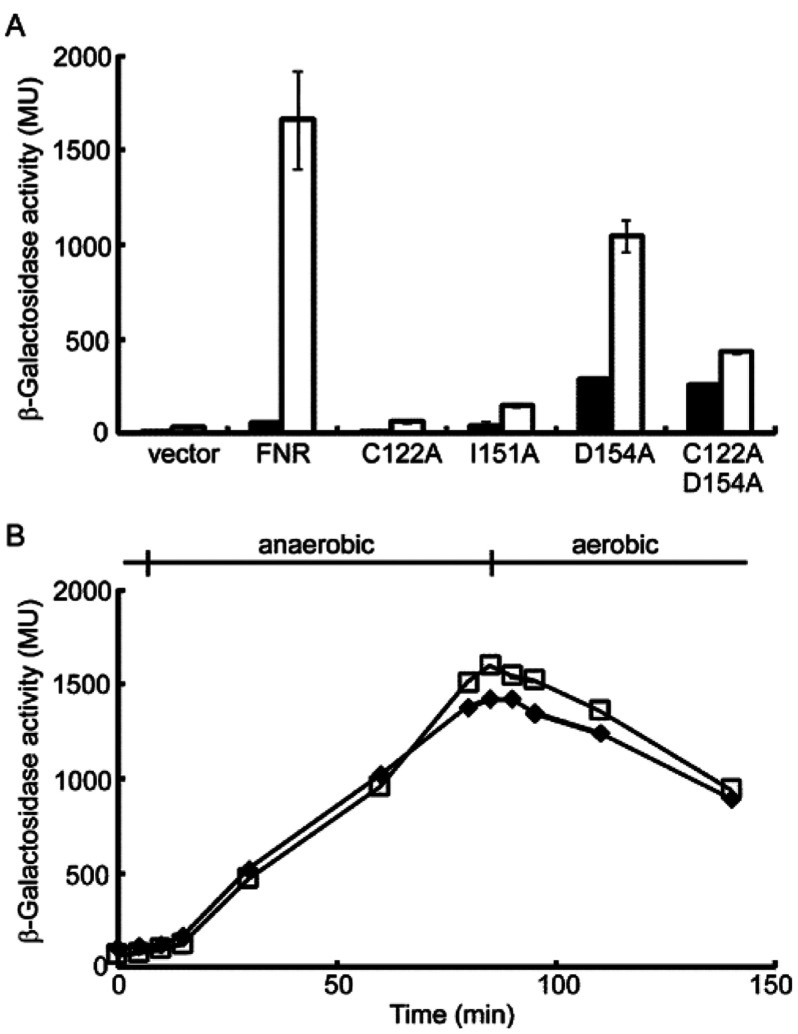
*In vivo* responses of FNR variants to O_2_ (**A**) Reporter gene (*lacZ*) expression driven from a single-copy chromosomal FNR-dependent synthetic promoter (FF-41.5) was measured for exponential-phase aerobic (closed bars) and anaerobic (open bars) cultures. The chromosomal copy of *fnr* was deleted from the host strain so that the regulatory properties of the indicated FNR variants could be estimated by measuring β-galactosidase activity (Miller units, MU). The data shown are means±S.D. for at least three independent experiments. (**B**) Dynamics of FNR and D154A FNR regulatory activity when O_2_ was withdrawn or introduced into *E. coli* cultures. Cultures were grown under aerobic conditions to mid-exponential phase before transfer to anaerobic conditions and then back to aerobic conditions. Throughout the experiment, samples were removed for measurement of β-galactosidase activity as a proxy for FNR activity. The β-galactosidase activities shown are corrected by subtraction of the activities obtained for C122A FNR from those for FNR and C122A/D154A FNR from 154A FNR to remove any non-iron–sulfur cluster-dependent changes. FNR, closed diamonds; D154A FNR, open squares. The data shown in (**B**) are typical of three independent experiments.

The activity of FNR is controlled by incorporation of an O_2_-sensitive [4Fe-4S] cluster into FNR monomers resulting in the formation of FNR dimers that exhibit enhanced site-specific DNA binding [3,8,12]. The FNR variants D154A and I151A are dimeric and monomeric respectively, irrespective of the presence or absence of the [4Fe-4S] cluster [17,31]. As expected, the *in vivo* activity of wild-type FNR was very low under aerobic conditions and was strongly enhanced (~30-fold) under anaerobic conditions (Figure 1A). The anaerobic activity of the C122A FNR variant, which lacks one essential iron–sulfur cluster co-ordinating cysteine residue, was close to that of the vector control, suggesting that this variant fails to acquire a [4Fe-4S] cluster *in vivo* (Figure 1A). I151A FNR resembled C122A FNR in that both had low activity under aerobic and anaerobic conditions, consistent with the inability to form homodimers, and confirming previous results using the *nar* promoter as a reporter of I151A FNR activity [17] (Figure 1A). In contrast, the aerobic activity of the D154A FNR variant was significant, presumably due to its homodimeric nature, and the enhancement in activity under anaerobic conditions (3.6-fold) was significantly less than that observed for wild-type FNR (*P*=0.042 in a two-tailed Student's *t* test), consistent with previous observations in which D154A FNR had only ~75% of the anaerobic activity of wild-type FNR (Fig-ure 1A) [3]. Analysis of the C122A/D154A FNR variant revealed only a 1.5-fold enhancement in activity under anaerobic conditions (Figure 1A). This suggests that the enhanced anaerobic activity of D154A FNR is due to iron–sulfur cluster acquisition and that the aerobic activity of this variant is due to its propensity to dimerize and consequently bind DNA.

The results described above and elsewhere are consistent with a series of events in which iron–sulfur cluster acquisition by wild-type FNR monomers is followed by dimerization and then site-specific DNA binding upon transfer from aerobic to anaerobic conditions. In contrast, the aerobic activity associated with D154A FNR indicates that it is pre-loaded on to the DNA, and upon transfer to anaerobic conditions, the incorporation of iron–sulfur clusters further enhances transcriptional activity by improving productive interactions with RNA polymerase [32] (Figure 1A). Therefore, the effect of the D154A substitution on the rate of FNR activation was tested *in vivo*. *E. coli* JRG6348 is an *fnr* mutant with a single-copy *lacZ* gene under the control of an FNR-dependent promoter (FF-41.5). Aerobic cultures of JRG6348 containing plasmids expressing FNR, C122A FNR, D154A FNR or C122A/D154A FNR, were grown to exponential phase and FNR activity was estimated by measuring β-galactosidase activity. The cultures were then transferred to anaerobic conditions and sampled at intervals to determine the rate of induction of FNR activity, before re-oxygenating the cultures by returning them to the shake flasks. The initial β-galactosidase activity for D154A FNR cultures was 3.5-fold greater than that for wild-type FNR, consistent with target DNA binding by the former, independent of O_2_ availability. To correct for the non-iron–sulfur cluster-dependent activity of D154A FNR, the values obtained for C122A FNR and C122A/D154A FNR, which are incapable of significant iron–sulfur acquisition, were subtracted from those of the FNR and D154A FNR cultures respectively. The data show that the responses of wild-type and D154A FNR proteins were superimposable (Figure 1B). Thus, there was no significant difference in the rate of β-galactosidase accumulation upon switching aerobic cultures to anaerobic conditions and vice versa (Figure 1B). Therefore, the results suggest that pre-dimerization/loading of apo-FNR on to target DNA does not affect the rate of iron–sulfur cluster-dependent β-galactosidase synthesis when cultures are transferred to anaerobic conditions. Similarly, decoupling iron–sulfur cluster disassembly from the FNR dimer–monomer transition did not significantly alter the output from the reporter (β-galactosidase) when anaerobic cultures were exposed to O_2_.

### Cluster incorporation into C16A/C122A, D154A and I151A FNR variants

**Figure 2:**
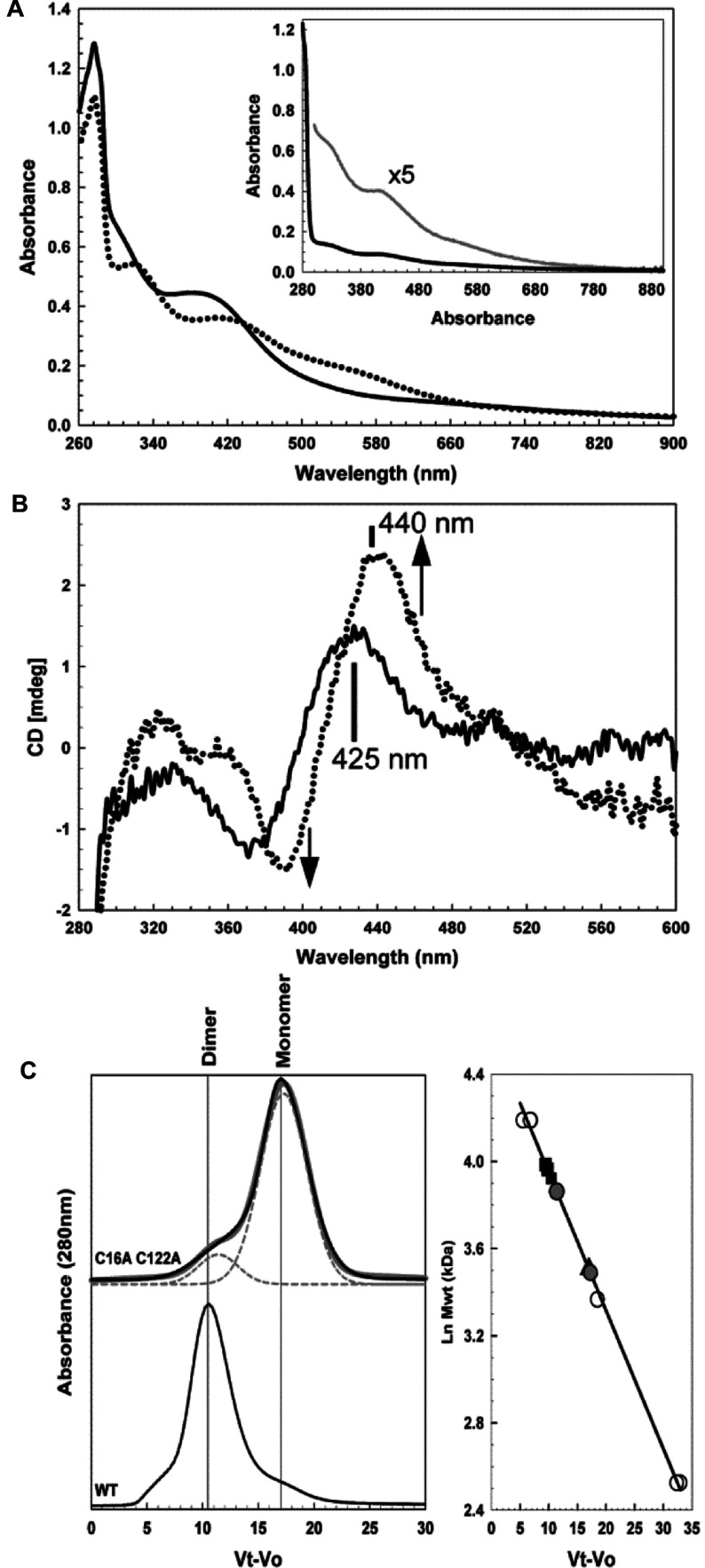
Optical properties of C16A/C122A FNR (**A**) Absorbance spectra and (**B**) CD spectra of reconstituted C16A/C122A FNR (~27 μM [4Fe-4S], 40% replete). In the presence (broken line) and absence (continuous line) of a 2-fold excess of O_2_. Inset, absorption spectrum of C16A/C122A FNR isolated under anaerobic conditions (black line), revealing the presence of a small amount (~4% replete) cluster (grey line). (**C**) The left-hand panel shows gel-filtration chromatography of C16A/C122A FNR (415 μM [4Fe-4S]). In grey is a fit of the chromatogram to multiple Gaussian peaks, revealing peaks corresponding to masses of 48 kDa and 33 kDa, accounting for ~20% and 80% of the total peak area respectively. The chromatogram of predominantly dimeric wild-type FNR (~70 μM [4Fe-4S], 94% replete) is shown below for comparison. The right-hand panel shows a calibration curve for the Sephacryl S100HR column. Open circles correspond to standard proteins (BSA, carbonic anhydrase and cytochrome *c*), black squares correspond to [4Fe-4S] wild-type FNR (54 kDa), black triangles correspond to [2Fe-2S] wild-type FNR (33 kDa) and grey circles correspond to C16A/C122A FNR peaks. The buffer was 25 mM Hepes, 2.5 mM CaCl_2_, 100 mM NaCl and 100 mM NaNO_3_ (pH 7.5).

C122A FNR exhibited remarkably low activity *in vivo*, and we wished to know whether this was due to a deficiency in [4Fe-4S] cluster binding, in cluster-mediated dimerization or both. To determine this, FNR containing the C122A substitution was purified. This protein also contained a second substitution, in which Cys^16^, a fifth cysteine residue in FNR that is not involved in cluster co-ordination, was replaced with alanine. Previous studies showed that this cysteine residue can be substituted without affecting the activity of the protein [7]. Because it lies relatively close to the cluster in wild-type FNR, it was removed from here to ensure that it could not become a cluster ligand upon reconstitution of FNR containing the C122A substitution. Anaerobically isolated C16A/C122A FNR was almost colourless. The UV–visible absorbance spectrum of a concentrated sample revealed the presence of a small amount of an iron–sulfur cluster (~4% based on a typical [4Fe-4S] cluster molar absorption coefficient), demonstrating a deficiency in cluster incorporation *in vivo*. *In vitro* reconstitution of the cluster generated a sample containing an iron–sulfur cluster (at ~40% loading), with UV–visible absorbance properties characteristic of a [4Fe-4S] cluster (Figure 2A). Since iron–sulfur proteins derive their optical activity from the fold of the protein to which they are ligated, their CD spectrum can provide detailed information about the local cluster environment. The CD spectrum of C16A/C122A FNR was very different from that of wild-type FNR and, in general, was not typical of a [4Fe-4S] cluster, more closely resembling a blue-shifted version of a [2Fe-2S] cluster (Figure 2B). The cluster remained O_2_-sensitive, however, undergoing conversion into a more typical [2Fe-2S] form upon titration with air-saturated buffer (Figures 2A and 2B). The association state of the anaerobically reconstituted protein was examined by gel filtration (Figure 2C). Importantly, the sample exhibited behaviour distinct from that of the natively folded FNR dimer. A wild-type sample at a similar cluster loading (40%) would be expected to run as 60% monomer and 40% dimer. The C16A/C122A FNR sample ran as 80% monomer and 20% dimer (or close to dimer). This indicates that the capacity of [4Fe-4S] C16A/C122A FNR to form a stable dimer is much diminished.

Thus, in the absence of Cys^122^, FNR does not incorporate significant amounts of cluster *in vivo*, but can accommodate a cluster through *in vitro* reconstitution. However, this has unusual spectroscopic properties, and the protein does not efficiently dimerize. This suggests that the correct arrangement of cluster ligands is crucial for the formation of a stable dimeric form upon cluster incorporation. These *in vitro* studies are consistent with the *in vivo* properties of the C122A variant.

**Figure 3:**
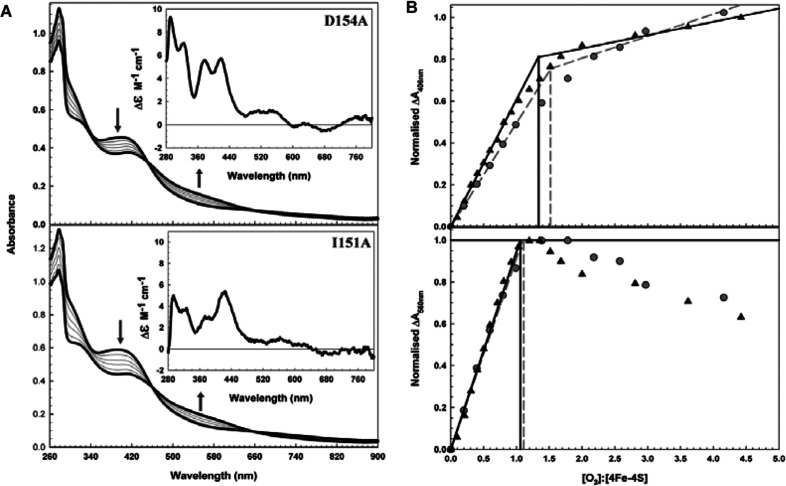
Oxidation of [4Fe-4S] D154A and I151A FNR proteins (**A**) UV–visible absorption titration of 28 μM [4Fe-4S] D154A and 36 μM [4Fe-4S] I151A FNR, as indicated, with buffer A containing dissolved atmospheric O_2_. The upper and lower spectra (bold, black) correspond to an [O_2_]/[4Fe-4S] ratio of 0 and ~1 respectively. Arrows indicate the movement of spectral features. The samples were incubated at 19°C for 5 min after each addition before measurement. Inset: CD spectra of FNR variants. Molar absorption coefficients relate to the [4Fe-4S] concentration. (**B**) Plots of normalized Δ*A*_420 _and Δ*A*_560_, as indicated, for D154A FNR (grey squares) and I151A FNR (black triangles) against the [O_2_]/[4Fe-4S] ratio. A clear end point to the titration was not observed at 420 nm because higher [O_2_]/[4Fe-4S] ratios promote [2Fe-2S] degradation, which contributes to the Δ*A*_420 _readings. In such a case, Δ*A*_560_, a wavelength specific for the [2Fe-2S] cluster, provides further information. The intercept of the initial slope with the upper asymptote at higher O_2_ levels reveals a reaction stoichiometry of ~1 [O_2_]/[4Fe-4S] cluster for D154A and I151A. Increasing the [O_2_]/[4Fe-4S] ratio above ~1.5 caused degradation of the [2Fe-2S] cluster as is evident from the decrease in 560 nm for D154A FNR and I151A FNR. The buffer was 25 mM Hepes, 2.5 mM CaCl_2_, 100 mM NaCl, 100 mM NaNO_3_ and 500 mM KCl (pH 7.5).

D154A and I151A FNR proteins isolated from anaerobic cultures displayed a straw brown colour consistent with the presence of an iron–sulfur cluster. Gel filtration confirmed that the association states of the proteins were as reported previously: D154A was dimeric and I151A was monomeric, irrespective of the cluster content of the protein (not shown) [17]. Anaerobic reconstitution yielded highly coloured proteins. UV–visible absorbance spectra (Figure 3A) contained absorption maxima at 320 nm and 405 nm, together with a broad shoulder at 420 nm. These are very similar to the spectrum of wild-type [4Fe-4S] FNR [13] (and indistinguishable from those of the proteins containing a cluster following anaerobic isolation, not shown). Both D154A and I151A FNR samples were EPR-silent, consistent with the cluster being in the [4Fe-4S]^2+^ oxidation state (not shown). The CD spectrum of D154A FNR, like that of wild-type FNR, contained six positive features at 291, 323, 378, 418, 510 and 548 nm [13] (Figure 3A, inset). In contrast, I151A FNR displayed a distinct CD spectrum dominated by the positive feature at 420 nm. Other positive features observed at 293, 323 and 375 nm were similar to those of wild-type FNR, with features at ≥500 nm being poorly resolved (inset Figure 3A). When compared with the wild-type and D154A spectra, it is apparent that the changes in the I151A spectrum arise from a loss of intensity from all the major bands apart from that at 420 nm. Moore and Kiley [17] showed that the far-UV CD spectrum of [4Fe-4S] I151A was indistinguishable from that of wild-type FNR, implying that the two proteins contain equivalent secondary structure content. Therefore, the observed changes are most probably due to subtle effects on the cluster caused by the difference in association state of I151A (monomeric) compared with wild-type and D154A FNR (both dimeric).

### D154A and I151A FNR variants undergo O_2_-mediated [4Fe-4S] into [2Fe-2S] cluster conversion

To determine whether the altered association state properties of the variants influenced their O_2_-sensitivities, [4Fe-4S] D154A and I151A FNR variants were titrated with air-saturated buffer (232 μM O_2_, 19°C). This resulted in a decrease in the absorbance at 420 nm and a concomitant increase in the 460–660 nm region, as reported for wild-type FNR (Figure 3A). Plotting Δ*A*_420_ against the [O_2_]/[4Fe-4S] ratio resulted in superimposable curves, suggesting that both clusters react in a similar way despite apparent differences in their local environment (Figure 3B). Clear end points to the titrations were not obtained, as for wild-type FNR, because partial degradation of the [2Fe-2S] cluster during the course of the titration contributes to the Δ*A*_420 _readings. This is apparent from plotting Δ*A*_560 _against the [O_2_]/[4Fe-4S] ratio (Figure 3B). The *A*_560 _ reading is specific to the [2Fe-2S] cluster and clearly shows complete formation of the [2Fe-2S] cluster in D154A, I151A and wild-type samples at [O_2_]/[4Fe-4S] ≈1.0, with subsequent loss of the [2Fe-2S] cluster at [O_2_]/[4Fe-4S] ratios above 1.5.

### Kinetic characteristics of the D154A and I151A FNR [4Fe-4S] into [2Fe-2S] conversion

**Figure 4:**
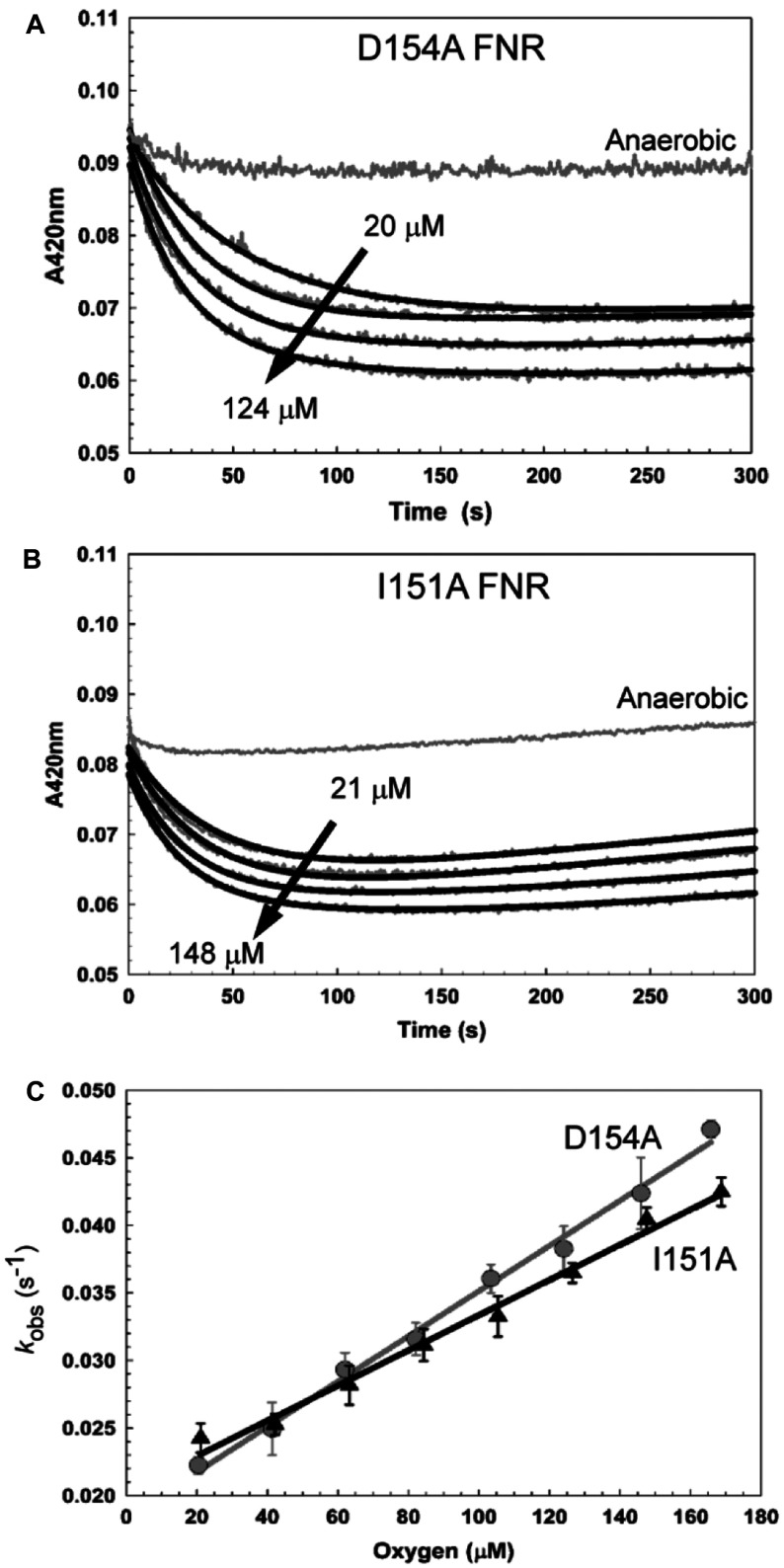
Rate of D154A and I151A FNR cluster conversion The rate of O_2_-dependent cluster conversion of 5 μM [4Fe-4S] (**A**) D154A and (**B**) I151A FNR was measured by absorbance at 420 nm. Traces shown were recorded at O_2_ concentrations of 0, 20, 62, 103 and 124 μM for D154A and 0, 21, 63, 126 and 148 μM for I151A FNR. Data are shown in grey; fits to the experimental data are black. (**C**) Plots of the first observed (first-order) rate constants obtained from the data in (**A**) for D154A (grey circles) and (**B**) I151A (black triangles) and additional experiments (at O_2_ concentrations of 41, 82, 146, and 166 μM for D154A and 42, 84, 105 and 169 μM for I151A FNR) as a function of the O_2_ concentration. Least squares linear fits of the data are drawn in for D154A (grey line) and I151A (black line). The gradients of these lines correspond to the apparent second-order rate constants.

The first step of wild-type FNR cluster conversion involves the O_2_-dependent reaction of [4Fe-4S]^2+^ to generate a [3Fe-4S]^1+^ intermediate [14]. This is followed by a spontaneous conversion of the intermediate into the [2Fe-2S]^2+^ form, co-ordinated by up to two cysteine persulfides [33]. D154A and I151A were exposed to different [O_2_]/[4Fe-4S] ratios, and the 420 nm decays were measured under pseudo-first-order conditions (Figures 4A and 4B). Datasets were then fitted as described in the Materials and methods section. Plots of *k*_obs_ against the O_2_ concentration for both D154A FNR and I151A FNR revealed a linear relationship, suggesting that the conversion from a [4Fe-4S]^2+^ into a [3Fe-4S]^1+^ cluster remains O_2_-dependent (Figure 4C). The apparent second-order rate constant, *k*_1_, for D154A was 167 (±5) M^−1^·s^−1^ and is comparable with that measured previously for wild-type FNR [180 (±10) M^−1^·s^−1^] in the same buffer (obtained from data reported in [13]). I151A FNR gave an apparent second-order constant of 130 (±6) M^−1^·s^−1^ (Figure 4C).

**Figure 5:**
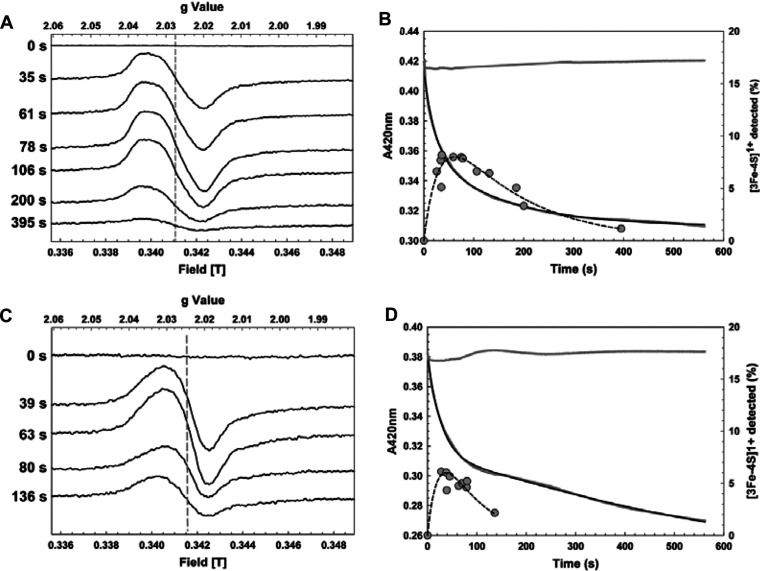
Detection of [3Fe-4S]^1+^ intermediate formation and decay by EPR and optical spectroscopies EPR spectra of reconstituted 23 μM [4Fe-4S] (**A**) D154A FNR and (**C**) I151A FNR in buffer A as a function of time after exposure to O_2_. EPR parameters: temperature, 15 K; microwave power, 2.0 mW; frequency, 9.67 GHz; modulation amplitude, 0.5 mT. Spectra are normalized to the same gain. The broken line indicates the shift in ***g***-value in the intermediate species. (**B** and **D**) *A*_420 _decay as a function of time following addition of O_2_ to D154A (**B**) and I151A (**D**) FNR, along with time-dependent EPR data (filled circles). Simultaneous exponential fits (see the ‘Data analysis’ section) of the optical and EPR data are shown as continuous and broken lines respectively.

Time-resolved EPR measurements were performed as described previously for FNR [13,14] to detect the formation and decay of the [3Fe-4S]^1+^ intermediate. Data for D154A are shown in Figure 5(A). A signal very similar in form to that of wild-type FNR was observed, maximizing at ~80 s before diminishing to a minor component at 395 s. Signals due to the [3Fe-4S]^1+^ intermediate were integrated and intensities were plotted as a function of time (Figure 5B). This demonstrates that the maximum signal corresponded to <10% of the starting [4Fe-4S] cluster concentration. This compares with ~30% for the wild-type protein [13,14]. An absorbance decay measurement was performed under conditions identical with that of the EPR experiment (Figure 5B), and both datasets were simultaneously fitted to a two-step process. This gave a pseudo-first-order rate constant for the formation of the [3Fe-4S]^1+^ intermediate of *k_1_*=0.038 (±0.002) s^−1^. Division by the O_2_ concentration (~220 μM) gives an estimate of the second-order rate constant, *k*=~172 M^−1^·s^−1^, consistent with the measurements presented above. The rate constant for the second step was *k*_2_=6.3 (±0.1) × 10^−3^ s^−1^, and the ratio of rate constants for the first and second steps, *k*_1_/*k*_2_, was ~6, somewhat lower than that for the wild-type protein (*k*_1_/*k*_2_=~7). This suggests that the second step occurs relatively more rapidly in D154A than in the wild-type protein, consistent with the detection of lower concentrations of the [3Fe-4S]^1+^ intermediate.

Equivalent experiments were performed for I151A (Figures 5C and 5D). In the present paper, signals due to the [3Fe-4S]^1+^ intermediate were also observed, maximizing at ~40–50 s, before decaying to a minor component by ~150 s. The form of the signal is somewhat different from that of wild-type, with a more isotropic shape and a ***g***-value centred on ***g*** ~2.025 (compared with ***g*** ~2.0275 for the wild-type FNR intermediate). This is consistent with differences in the [4Fe-4S] cluster environment detected by CD (see above). Signal integration indicated that the maximum intensity corresponded to only ~6% of the starting [4Fe-4S] cluster concentration. Fitting of EPR data gave rate constants for the formation and breakdown of the [3Fe-4S]^1+^ intermediate of *k*_1_=0.033 (±0.003) s^−1^ and *k*_2_=0.019 (±0.001) s^−1^ respectively, giving *k*_1_/*k*_2_=~1.7. Thus, the rate of decay of the [3Fe-4S]^1+^ species was enhanced significantly in this variant compared with wild-type and with D154A. It should be noted that a third exponential was needed to fit the UV–visible absorbance data, with rate constants for the first two processes matching those above and a third rate constant of 1.5 (±0.6) × 10^−4^ s^−1^. This corresponds to the decay of the [2Fe-2S] cluster to the apo-form (see below). Since both [2Fe-2S]- and apo-forms are EPR-silent, this reaction does not contribute to the EPR signal. A third exponential was not required to fit the D154A data (or that of wild-type [14]), because the formation of the [2Fe-2S] form was slower and significant decay of the [2Fe-2S] form did not take place during the timescale of the measurement.

### O_2_-mediated loss of the FNR [2Fe-2S] cluster is unaffected by association state

**Figure 6:**
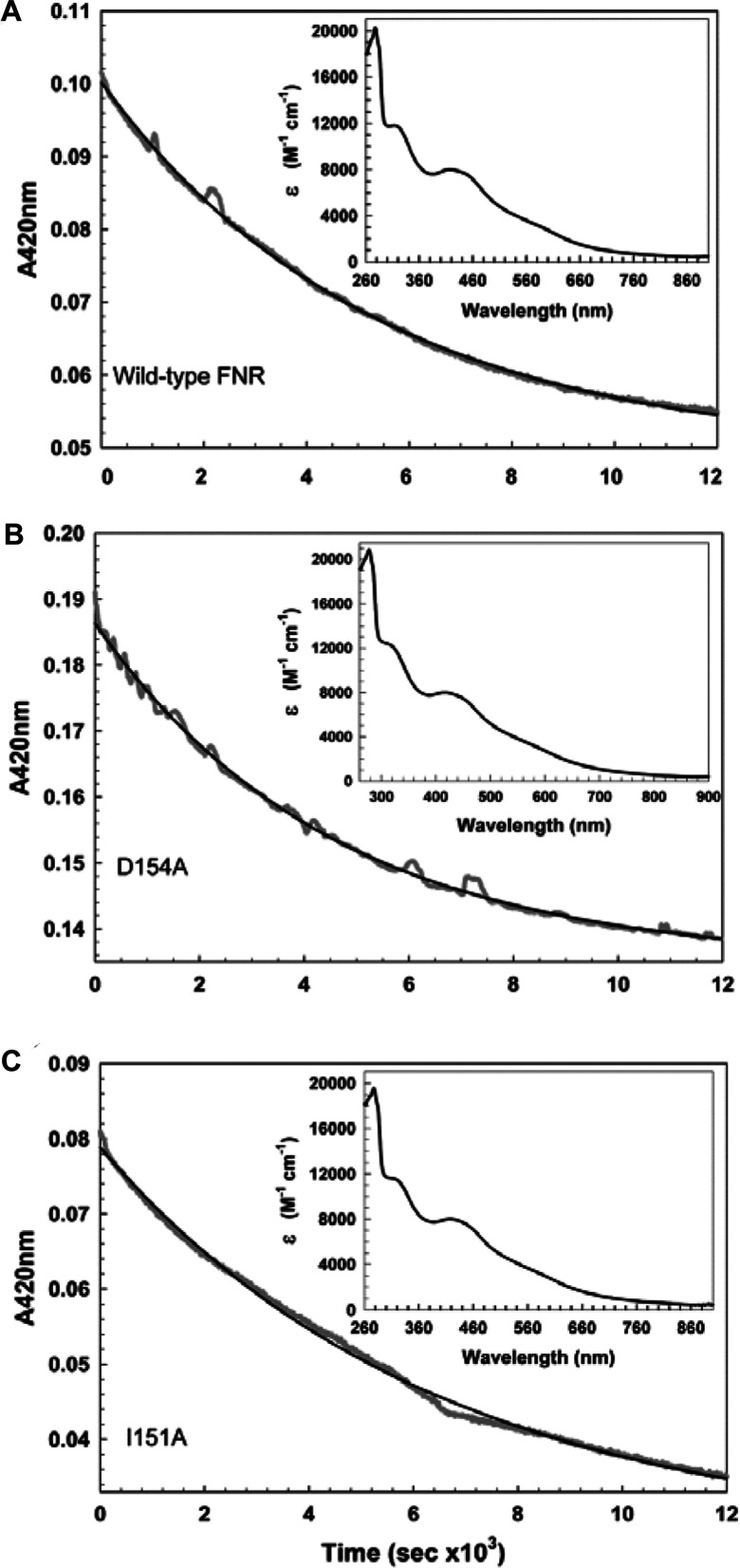
Stability of [2Fe-2S] forms of I151A and D154A FNR Plots of *A*_420_ against time for aerobic samples of [2Fe-2S] forms of (**A**) wild-type FNR (13 μM); (**B**) D154A FNR (23 μM); and (**C**) I151A FNR (13 μM). The data (grey lines) were fitted to a single exponential function (black lines, see the ‘Data analysis’ section) yielding pseudo-first-order rate constants. Inset: absorption spectra of isolated [2Fe-2S] wild-type, D154A FNR and I151A FNR respectively.

To investigate the relative stabilities of the converted cluster, [2Fe-2S] forms of wild-type, D154A and I151A FNR proteins were generated and then purified under anaerobic conditions (see the Materials and methods section). Protein solutions were mixed with aerobic buffer, and changes in *A*_420 _were monitored. The resulting data (Figure 6) fitted well to a single exponential function, consistent with the conversion of [2Fe-2S] FNR into apo-FNR, with *k*_obs_ values of 2.2 (±0.01) × 10^−4^, 1.6 (±0.01) × 10^−4^ and 1.9 (±0.01) × 10^−4^ s^−1^ for D154A, I151A and wild type respectively. Thus, there are only relatively small differences in reactivity of the [2Fe-2S] forms towards O_2_.

### FNR bound to DNA exhibits an enhanced rate of [4Fe-4S] into [2Fe-2S] conversion

**Figure 7:**
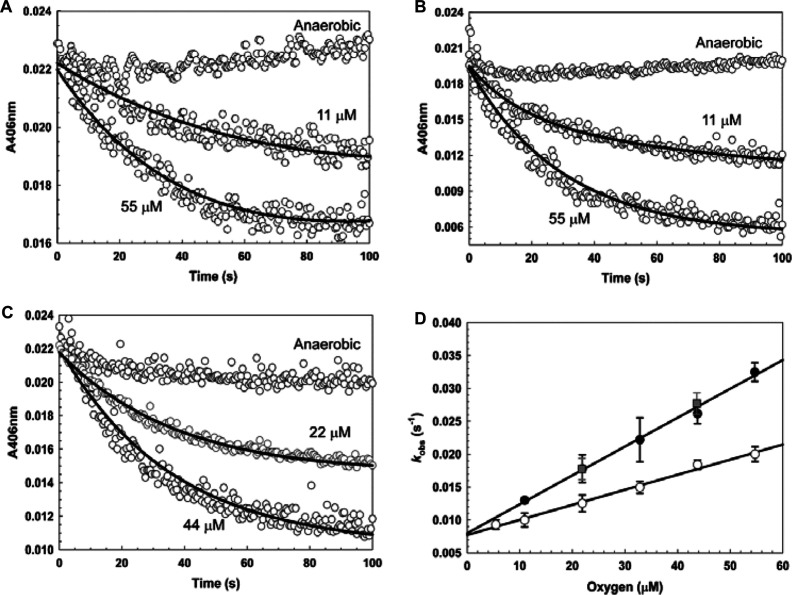
Kinetics of wild-type FNR cluster conversion in the presence of DNA The rate of O_2_-dependent cluster conversion of (**A**) wild-type FNR (~1 μM [4Fe-4S] FNR, equivalent to ~0.5 μM dimer); (**B**) as (**A**) but in the presence of pGS422 (~0.6 μM); (**C**) as (**A**) but in the presence of a 345-bp FF-41.5 DNA fragment (~0.6 μM) was measured by absorbance at 406 nm. Reactions at two O_2_ concentrations are shown for each condition. Data (shown in grey) are averages of three measurements; fits to the experimental data are in black. (**D**) Plots of the first observed (first-order) rate constants obtained from the data in (**A**–**C**) and additional experiments for FNR (white circles), FNR in the presence of pGS422 (black circles), and FNR in the presence of a 345-bp FF-41.5 fragment (grey squares) as a function of the O_2_ concentration. Least squares linear fits of the data are drawn in for FNR and FNR in the presence of pGS422, the gradients of which correspond to the apparent second-order rate constants. The buffer was 20 mM Tris/HCl and 5% glycerol, pH 8.0.

To investigate the effects of DNA on the kinetics of cluster conversion, anaerobic wild-type [4Fe-4S] FNR was mixed with aliquots of pGS422, a plasmid containing a consensus (TTGATGTACATCAA) FNR-binding site, in buffer C (see the Materials and methods section), to give a 1.2-fold excess of DNA. Wild-type FNR was shown previously to bind specifically (*K*_d_ ~14 nM) to the FF-41.5 promoter in anaerobic buffer in an O_2_-dependent manner [34,35]. In the absence of pGS422, cluster conversion occurred in an O_2_-dependent manner under pseudo-first-order conditions, as observed previously [14]. A double exponential function was needed to fit the data. The second phase of the reaction, which only begins to contribute significantly towards the end of 100 s acquisition period, corresponds to a slow increase in absorbance; this is unusual, but has been observed previously under certain conditions [13] and is believed to be associated with the instability of the [3Fe-4S]^1+^ intermediate, or with the propensity of ejected Fe^2+^ to precipitate. Despite this, the rate constant for the initial step can be readily obtained [13]; the apparent second-order rate constant under these conditions (in buffer C) was *k*_1_=229 (±10) M^−1^·s^−1^ (Figures 7A and 7D). In the presence of pGS422, the datasets were best described by a single exponential function (Figure 7B), indicating that the conversion of the [3Fe-4S]^1+^ intermediate into the [2Fe-2S] form occurs more rapidly, such that the two steps of cluster conversion cannot be easily distinguished when FNR is DNA-bound. Similar observations were made previously for FNR in the presence of a Fe^3+^ chelator [13]. Plotting *k*_obs_ obtained in the presence of pGS422 against the O_2_ concentration revealed a linear dependence on O_2_, with an apparent second-order rate constant, *k*_1_=439 (±28) M^−1^·s^−1^, approximately twice that of FNR in the absence of pGS422 (Figure 7D). Experiments using a 345-bp FF-41.5 linear DNA fragment in place of pGS422 gave results very similar to those obtained with supercoiled plasmid DNA (Figures 7C and 7D).

## DISCUSSION

FNR and members of the CRP family are transcriptional regulators that bind as dimers to DNA operator sequences. *E. coli* FNR is unusual in undergoing a monomer–dimer conversion on switching between bound and unbound states, whereas other characterized family members maintain a dimeric state in both forms. It is the binding of a [4Fe-4S] cluster to FNR that leads to protein dimerization, thus promoting specific high-affinity DNA binding. Reaction of the [4Fe-4S] cluster with O_2_ generates a [2Fe-2S] form, leading to monomerization, with loss of high-affinity DNA binding. Since the presence of the [4Fe-4S] cluster controls the wild-type protein's association state, and hence its DNA-binding characteristics, we have proposed that the inability to dimerize following cluster binding, or the inability to monomerize in the presence of O_2_, might significantly influence the [4Fe-4S] cluster reactivity. To test this, we have used two variants of FNR in which the association state is essentially decoupled from the form of the cluster.

A molecular model based on the high-resolution structure of CRP indicates that Asp^154^ is located at the interface of the dimerization helices, the negative charge associated with this residue preventing dimerization [17]. Binding of a [4Fe-4S] remotely from this site causes a conformational rearrangement that shields the negative charge, enabling dimerization. Substitution of alanine for Asp^154^ removes the negative charge, leading to dimerization even in the absence of cluster-dependent structural changes. Thus, D154A FNR remains predominantly dimeric even in the presence of O_2_. Ile^151^ is predicted to lie below D154A also in the region of the dimerization helices [17], where it is proposed to play a key role in shielding the negative charge due to Asp^154^ when FNR binds a [4Fe-4S] cluster. Substitution of alanine for this residue results in ineffective shielding, such that dimerization cannot occur. Thus, I151A FNR is monomeric, even in a cluster bound form. Analysis of FNR containing the C122A substitution, which is deficient in cluster incorporation and hence dimerization, together with these two variants confirmed the importance of both dimerization and iron–sulfur cluster acquisition for FNR activity *in vivo*.

Consideration of the dynamics of FNR switching *in vivo* showed that the dimeric variant, FNR D154A, was activated for gene expression at a rate comparable with that of wild-type FNR. Thus, it was concluded that dimerization is neither rate-limiting for FNR-dependent gene expression, nor is iron–sulfur cluster acquisition significantly impaired when the dimeric state of FNR is maintained, resulting in pre-loading FNR on to target DNA, in the presence or absence of O_2_ *in vivo*. Moreover, inactivation of FNR-regulated gene expression by O_2_ exposure was not impaired for FNR D154A *in vivo*. These data suggest that the reaction of the FNR [4Fe-4S] cluster with O_2_ and the rate of iron–sulfur cluster acquisition are not significantly altered by stabilizing the dimeric state of FNR *in vivo*, at least as far as FNR-dependent gene expression is concerned. Therefore, it can be suggested that the FNR monomer–dimer transition has evolved to minimize transcriptional activity in the presence of O_2_, as it appears that a dimeric apoprotein, mimicked by FNR D154A, would have significant DNA-binding and transcriptional activity, even in the absence of the [4Fe-4S] cluster. A detailed *in vitro* analysis of the properties of the O_2_ reactivities of the monomeric FNR I151A and dimeric FNR D154A proteins was undertaken to determine how the *in vivo* characteristics of these proteins relate to the properties of their iron–sulfur clusters.

The [4Fe-4S] cluster bound to FNR D154A was spectroscopically essentially identical with that of wild-type FNR. Its reaction with O_2_ was also similar, exhibiting only small differences. The overall conversion process was the same, involving an O_2_-dependent first step, generating a [3Fe-4S]^1+^ intermediate, which subsequently decayed to form a [2Fe-2S]^2+^ form. The latter step occurred somewhat more rapidly than in wild-type FNR, suggesting that the [3Fe-4S]^1+^ intermediate of D154A FNR is slightly less stable. Although the absorption spectrum of FNR I151A indicated that the [4Fe-4S] cluster is similar to that of the wild-type protein, the CD spectrum revealed significant differences, particularly with respect to intensities. Therefore, the local environment of the cluster is perturbed in comparison with wild-type and D154A FNR, suggesting a structural communication between the dimerization helix and the cluster-binding domain. The reaction of I151A with O_2_ is broadly similar to that of wild-type FNR, but is more significantly affected than that of D154A FNR. The rate constant for the initial reaction is similar to that of wild-type protein, whereas that for the second step is higher, with the consequence that the [3Fe-4S]^1+^ intermediate is not readily detected. This suggests that the [3Fe-4S]^1+^ is less stable in this monomeric form of FNR than in the wild-type protein. The slow decay of the [2Fe-2S] forms of D154A FNR and I151A FNR to apoprotein was similar to that of the wild-type protein.

The O_2_-reactivity data are consistent with a previous study that aerobic isolation of D154A FNR results in a cluster-free protein, suggesting that the protein retained an O_2_-sensitive cluster [10]. It is also consistent with the idea that the immediate cluster environment plays a key role in determining its reactivity with O_2_ [36,37], as the properties of the D154A FNR cluster are identical with those of the wild-type protein. For I151A, the modified cluster environment is consistent with the distinct reactivity. The increased rates of decay of the [3Fe-4S]^1+^ intermediates of D154A and (particularly) I151A can be rationalized by considering that the [3Fe-4S]^1+^ into [2Fe-2S]^2+^ conversion step is the one that most probably drives the monomerization process in wild-type FNR. In the permanent dimer D154A, this monomerization does not occur because the charge repulsion due to Asp^154^ upon cluster conversion is absent. Thus, the cluster conversion occurs more readily. In the permanent monomer I151A, there is no additional charge repulsion upon cluster conversion because the protein is already monomeric. Hence, again, the conversion process can occur more readily.

In view of contrasting literature reports and an evolving mechanistic understanding, we also wished to examine further the effect of DNA binding on cluster reactivity with O_2_ using DNA with an FNR promoter and all other possible *cis*-acting regulatory sequences. Addition of O_2_ led to the same overall reaction as in the absence of DNA, generating a [2Fe-2S]^2+^ cluster form. However, the decay data at 420 nm fitted well to a single exponential function, rather than a double exponential function (required to fit FNR data in the absence of DNA), suggesting that conversion of the [3Fe-4S]^1+^ intermediate into the [2Fe-2S]^2+^ cluster is enhanced such that the whole process appears to be a single-step reaction. The rate constant for the O_2_-dependent conversion reaction is ~2-fold higher for FNR when bound to DNA. This result is different from that reported previously for *E. coli* FNR bound to a 34-bp dsDNA fragment containing the FNR consensus sequence, for which no significant effect was found [18]. Our data are, however, at least in part similar to that reported for *Nm*-FNR. Studies of the effects of *Nm-*FNR binding to a 40-bp dsDNA fragment containing an FNR-controlled promoter showed that the rate constant for the initial O_2_-dependent conversion of [4Fe-4S] *Nm*-FNR into the [3Fe-4S] intermediate was doubled when *Nm-*FNR was DNA-bound compared with free in solution [19]. However, it was found that the [3Fe-4S]^1+^ intermediate species was subsequently stabilized against conversion into the [2Fe-2S]^2+^ form when *Nm-*FNR was DNA-bound [19]. In the present study, for *E. coli* FNR, the decay of the [3Fe-4S]^1+^ was found to be accelerated rather than stabilized.

These findings show that DNA-bound [4Fe-4S] FNR is the preferred target of O_2_, over the non-DNA-bound form. This would increase the O_2_-sensitivity of the FNR regulatory system because transcriptionally active [4Fe-4S] FNR would respond first to cytoplasmic O_2_ availability and, thus, maximize the sensitivity of the regulatory system.
